# Randomised clinical trial: the efficacy of prucalopride in patients with chronic intestinal pseudo-obstruction - a double-blind, placebo-controlled, cross-over, multiple *n* = 1 study

**DOI:** 10.1111/j.1365-2036.2011.04907.x

**Published:** 2011-11-08

**Authors:** A V Emmanuel, M A Kamm, A J Roy, R Kerstens, L Vandeplassche

**Affiliations:** * University CollegeLondon, UK; † St Vincent's Hospital & University of Melbourne, Melbourne, Australia, and Imperial CollegeLondon, UK; ‡ Shire-Movetis NVTurnhout, Belgium

## Abstract

**Background:**

Chronic intestinal pseudo-obstruction is a disabling condition for which there are no established drug therapies. The condition is caused by a diverse range of intestinal myopathies and neuropathies.

**Aim:**

To assess the therapeutic efficacy of prucalopride, a selective high-affinity 5-HT_4_ receptor agonist, we employed a multiple *n* = 1 study design. Each patient acted as his/her own control, each day counting as one treatment episode, allowing comparison of 168 days on each of active drug and placebo.

**Methods:**

Double-blind, randomised, placebo-controlled, cross-over trial of four 12-week treatment periods, with 2–4 mg prucalopride or placebo daily. In each of the first and second 6 months there was a prucalopride and a placebo treatment. Patients with proven chronic intestinal pseudo-obstruction, including dilated gut, were included. Evaluation was by patient diary and global evaluation.

**Results:**

Seven patients participated (mean 42 years, five female, median symptom duration 11 years). Three discontinued, two due to study length, and one on prucalopride due to unrelated malnutrition and bronchopneumonia. Four patients (three visceral myopathy and one visceral neuropathy) completed the study; prucalopride significantly improved pain in three of four patients, nausea in two, vomiting in one, bloating in four and analgesic intake. Bowel function was not changed substantially.

**Conclusions:**

*n* = 1 studies in rare conditions allow drug efficacy assessment. Prucalopride relieves symptoms in selected patients with chronic pseudo-obstruction.

## Introduction

The term chronic idiopathic intestinal pseudo-obstruction (CIP) denotes a clinical syndrome of apparent bowel obstruction in the absence of an obstructing lesion. The term ‘pseudo-obstruction' was first used to describe a cohort of patients with recurrent vomiting, abdominal pain and distension, who had undergone repeated laparotomy with no obstructive cause demonstrated.^1^ The symptoms relate in part to the failure of intestinal propulsion, due to an underlying disorder of enteric nerves or muscle.

The underlying pathology of this condition is heterogeneous, encompassing a range of visceral myopathies and neuropathies.^2^–^4^ The gut pathology may be primary (idiopathic) or secondary to a more widespread systemic disease of a degenerative, inflammatory, ischaemic or autoimmune nature.

The condition is rare with a wide spectrum of severity. It is sometimes diagnosed in the setting of abnormal gut motor function and pain without gut morphological change.^5^, ^6^ The diagnosis is definite when the gut is dilated, there is no obstructing lesion, and gut pathology has been established.

At present, there are no drug therapies proven in controlled trials to improve gut function or symptoms in this condition. Abdominal pain is a major symptom for many patients, and some ultimately require regular opiate analgesia. Nausea, vomiting and distension are almost invariable, culminating in the majority of patients requiring nutritional support. In many, the severity of symptoms leads with time to the need for home-based parenteral nutrition.^3^ The prokinetic, cisapride, has been studied in patients with CIP, and was modestly better than placebo in improving symptoms in adults.^7^ In an uncontrolled study, erythromycin, a motilin analogue, caused a decrease in vomiting and improved bowel function in some patients with primary and secondary CIP.^8^

Prucalopride, a substituted benzamide with selective 5HT_4_-agonist activity has been shown previously to improve symptoms in patients with idiopathic constipation, accelerating both upper and lower gut transit.^9^–^11^ A recent multi-centre randomised placebo-controlled study has shown that the drug improves bowel function and symptom burden in severe chronic constipation.^12^ The drug is well tolerated, with no significant increase in the number adverse effects compared with placebo.^10^, ^13^ Most recently, prucalopride has been approved in Europe for the symptomatic treatment of chronic constipation in women in whom laxatives fail to provide adequate relief.

The present study aimed to evaluate the efficacy and safety of prucalopride, in a once daily dose of 2–4 mg, in patients with known CIP. It was felt that, given the rarity of the condition and the diversity of the underlying pathology, it would not be possible to undertake a standardised parallel-group, placebo-controlled study. A parallel group study may also have obscured benefit for patients with some subtypes of pathology, while the group as a whole has a diversity of underlying pathologies which may respond differently to active treatment. The chronic and fluctuating nature of symptoms also means that any study needs to be performed over a long enough time period to demonstrate a difference between active treatment and placebo. To overcome these problems, we chose to undertake an ‘*n* = 1', placebo-controlled study in which individual patients acted as their own controls. Patients were studied over 48 weeks, receiving either prucalopride or placebo given once a day for four randomised periods of 12 weeks.

## Materials and Methods

### Trial design

We undertook a single-centre, randomised, double-blind, placebo-controlled, cross-over study in subjects with definite, established CIP. Patients were treated for periods of 12 weeks with either placebo or prucalopride once daily. Subject numbers were assigned in consecutive order and the following four sequences were used: ABAB, BABA, ABBA and BAAB (A = placebo, B = prucalopride). Active treatment consisted of a single 2 mg tablet of prucalopride. If patients felt one tablet was not providing symptomatic relief, they could increase to two tablets according to subjective response and after confirmation with the investigators. If they experienced side-effects, down titration back to one tablet was allowed. In each of the first and second 6 month periods there was one placebo and one active drug treatment period. There were no wash-out periods. Study visits took place at weeks 0, 4, 12, 16, 24, 36 and 48 of treatment. Patients kept a daily symptom diary to collect daily symptom severity scores.

### Patients

Seven patients (five female, median age 39) were enrolled into the study. They had long-standing, definite CIP with a median duration of illness of 137 months. To be included, patients had to have had episodic obstructive symptoms and radiological evidence of small bowel dilatation, in the proven absence of obstructing pathology. All patients had gut tissue confirmation of their underlying gut pathology: four patients had primary visceral neuropathy, two had primary visceral myopathy, and one had visceral myopathy secondary to scleroderma. All subjects were on stable doses of medication for their CIP. If the subject was already taking a prokinetic drug this could be continued, recording daily intake in the trial diary. Subjects could not, however, start on new medication during the study. Table 1 shows the clinical data.

**Table 1:** Patient demographics

Parameter	All patients (*n* = 7)
	Number
Sex	
Female	5
Male	2
Primary disease	
Visceral myopathy	3
Visceral neuropathy	4
	Median (Min; Max)
Age, years	39 (28; 68)
Body mass index	21.7 (17.5; 23.6)
Weight, kg	50 (42; 68)
Time since onset of symptoms, months	137 (80; 160)

The study was approved by the Northwick Park and St Mark's Ethics Committee. All subjects gave written informed consent to participate in the study. Trial registration number: ClinicalTrials.gov NCT007 93247.

## Efficacy

Patients recorded the following data on a daily basis in their diary:

- Intake of medication.
- Time of bowel emptying.
- Consistency of stools (watery, paste, normal, hard or very hard).
- Time of intake of laxatives and analgesics.
- Intake and amount of erythromycin.
- The occurrence and severity of each of four symptoms: pain, nausea, vomiting and bloating, each scored as 0 = absent, 1 = mild, 2 = medium, 3 = severe, or 4 = could not be worse.
- The ability to eat food and finish a meal (yes/no).

### Safety and adverse events

Patients recorded any adverse effects in their daily diary, and reported any to the investigator. The investigator determined how likely it was that the reported adverse effect was related to study medication. Due to the nature of CIP, it was almost inevitable that patients would be admitted to hospital during the course of a 1-year study. Admissions for severe episodes of CIP were noted, but not reported as adverse events.

Blood samples for biochemistry, haematology and urinalysis, were performed at weeks 0 and 24. Blood pressure, heart rate and ECG were assessed at week 0 and 24.

### Statistical analysis

As a multiple *n* = 1 study, descriptive statistics and graphical visualisation of results for each patient over time were considered as most important for the evaluation of efficacy and safety parameters. For each patient these data of the different 12 week treatment periods were summarised per treatment: placebo or prucalopride analysed as the total for each period of 12 weeks.

The primary endpoint was the proportion of days that each of the main symptoms was present (i.e. score >0). Secondary end-points included the mean score for each symptom. The primary comparison was always a within- subject comparison of results during treatment with prucalopride vs. results during placebo treatment. For each subject, the Wilcoxon rank sum test was used to compare the daily symptoms severity scores during prucalopride treatment with the scores during placebo treatment.

## Results

### Patients

Seven patients (four visceral neuropathy and three visceral myopathy) entered the study. Three patients, all with visceral neuropathy, discontinued treatment shortly after the first treatment period: one due to serious adverse events believed to be unrelated to the study medication (feeding line infection, sepsis, bronchopneumonia and malnutrition), and two due to withdrawal of consent (one each on placebo and prucalopride) as the individuals felt unable to comply with maintaining a daily diary. The other four subjects (three visceral myopathy and one visceral neuropathy) completed the four treatment periods and had sufficient symptom scores available for evaluation of efficacy.

Table 2 shows the treatment details for each subject. Of patients completing the study, three used mainly a daily dose of 2 mg, and one patient used a daily dose of 3 mg of prucalopride on average during the active drug periods.

**Table 2:** Treatment details

Patient	Phase	Treatment	Mean daily dose, mg (Inclusive days off drug)	Treatment duration, days
A03001 (vn)	Period 1	Pru	3	99
A03002 (vm)	Period 1	Pla	0	82
	Period 2	Pru	1.42	68
	Period 3	Pru	2	83
	Period 4	Pla	0	117
A03003 (vn)	Period 1	Pla	0	75
A03004 (vn)	Period 1	Pru	2	85
	Period 2	Pla	0	28
A03005 (vn)	Period 1	Pla	0	92
	Period 2	Pru	1.6	47
	Period 3	Pru	2	75
	Period 4	Pla	0	92
A03006 (vm)	Period 1	Pla	0	99
	Period 2	Pru	3	91
	Period 3	Pla	0	98
	Period 4	Pru	3	85
A03007 (vm)	Period 1	Pru	2	86
	Period 2	Pla	0	70
	Period 3	Pru	2	85
	Period 4	Pla	0	85

Pla, Placebo; Pru, Prucalopride; vm, visceral myopathy; vn, visceral neuropathy.

### Efficacy parameters

#### Symptom evaluations

**Figure 1:**
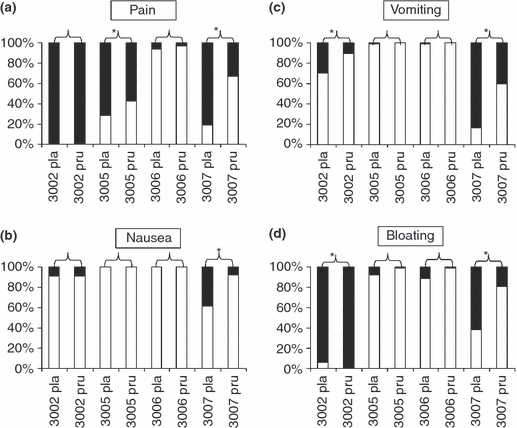
Proportion of days with symptoms (dark) vs. no symptoms (light) for (a) pain, (b) nausea, (c) vomiting and (d) bloating for each of the four patients completing the study. Each number (pair of bars) represents a different patient, for whom all days on placebo and active drug have been summed. *Statistically significant reduction (*P* < 0.05) of symptoms with prucalopride vs. placebo. Pru, Prucalopride; Pla, Placebo.

Figure 1 and Table 3 show the distribution of the proportion of days with and without symptoms (pain, vomiting, nausea and bloating) for each of the four patients who completed the study, during placebo- and prucalopride- treatment days. One subject (3006) reported very few days with the presence of symptoms during both placebo and prucalopride treatment, hence there was little room for improvement. All the other subjects showed an improvement in at least one of the symptoms with active treatment.

**Table 3:** Mean symptom severity score for each of the four patients completing the study

		Symptom							
		
		Pain		Vomiting		Nausea		Bloating	
					
Patient		PLA	PRU	PLA	PRU	PLA	PRU	PLA	PRU
A03002 vm	*N* (days)	176	142	169	142	170	142	171	141
	Mean	1.6	1.4	0.3	0.2	0.7	0.2	1.5	1.3
	s.d.	0.63	0.55	0.97	0.52	1.08	0.61	0.73	0.5
	*P*-value	<0.0001		0.7351		<0.0001		0.0092	
A03005 vn	*N* (days)	170	119	168	118	168	118	168	118
	Mean	0.9	0.7	0	0	0	0	0.1	0
	s.d.	0.68	0.72	0	0	0.08	0	0.27	0.09
	*P*-value	0.0391		1.000		0.4060		0.0080	
A03006 vm	*N* (days)	182	167	182	167	182	167	182	166
	Mean	0.1	0	0	0	0	0	0.1	0
	s.d.	0.35	0.17	0	0	0	0.08	0.31	0.08
	*P*-value	0.1656		1.000		0.2999		<0.0001	
A03007 vm	*N* (days)	146	163	141	161	146	161	143	160
	Mean	2.2	0.8	1.0	0.1	2.0	0.7	1.5	0.4
	s.d.	1.4	1.19	1.42	0.48	1.3	0.99	1.47	0.8
	*P*-value	<0.0001		<0.0001		<0.0001		<0.0001	

vm, visceral myopathy; vn, visceral neuropathy; s.d., standard deviation.

(0 = absent, 1 = mild, 2 = medium, 3 = severe, 4 = could not be worse).

Bloating improved in all subjects, with both the frequency and average severity decreasing with prucalopride in all subjects (Table 3). Vomiting was present in two subjects, with the average severity decreasing in both, and the frequency decreasing in one.

**Figure 2:**
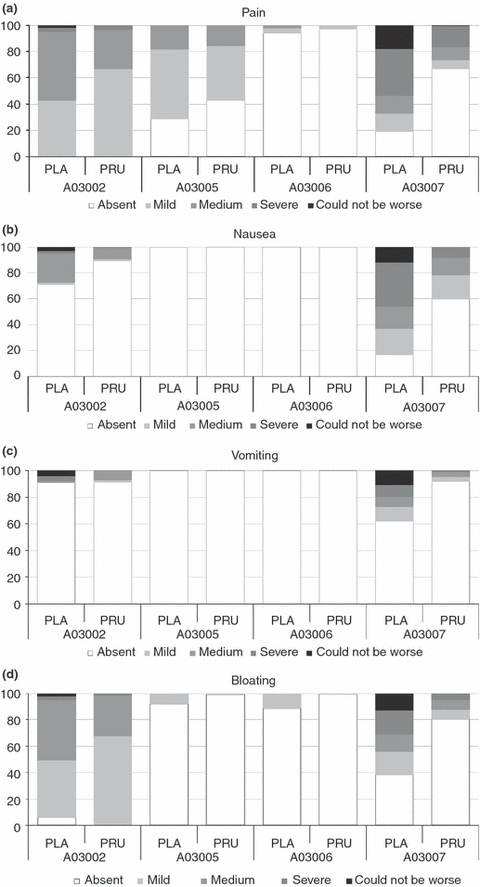
Proportion of days with each symptom severity score for (a) pain, (b) nausea, (c) vomiting and (d) bloating. Each pair of bars represents a different patient, for whom all days on placebo and active drug have been summed – 168 days on each of prucalopride and placebo treatments.

Symptoms changed in both frequency (Fig. 1) and severity (Fig. 2) with active treatment. Figure 2 shows all symptom severity scores for each patient, on prucalopride and placebo. Changes in symptom frequency did not always mirror changes in severity. For example in one patient (3002), whose treatment sequence was placebo-prucalopride-prucalopride-placebo, pain was reported for all days throughout the study (Fig. 1), but pain severity decreased significantly with prucalopride compared with placebo treatment (Table 3, Fig. 2).

Prucalopride did not affect the mean weekly stool frequency and consistency, the pattern of laxative use, the percentage of days able to eat food and finish a meal, and the number of periods of more than 24 h without a stool per 4 weeks.

##### Rescue analgesia use

Two subjects used on-demand (rescue) analgesia during the study. For both subjects, the number of analgesia intakes decreased substantially during treatment with prucalopride compared with the placebo periods (Table 4).

**Table 4:** Average weekly number of analgesia intakes per week in the two patients who used on-demand (rescue) analgesia during the study

		Analgesia			
		
Patient	Treatment	Number of intakes	Number of days with intakes	Average intakes/week (s.d.)	*P*-value (*t*-test)
A03002	Placebo	412	195	14.8 (11.03)	<0.001
	Prucalopride	211	169	8.7 (3.31)	
A03007	Placebo	370	149	17.4 (10.21)	<0.001
	Prucalopride	229	166	9.7 (6.24)	

s.d., standard deviation.

#### Body weight

Body weight was stable for all four patients during the course of the yearlong study.

### Adverse events

Three patients reported adverse events. One patient reported this during placebo treatment only. Another patient reported adverse effects during placebo and prucalopride treatment periods, and another during one prucalopride treatment period only.

All but one adverse event were experienced as severe, but none were judged by the investigator to be drug-related. The commonest complaints were of abdominal pain, constipation and vomiting.

One subject permanently stopped the trial medication and prematurely discontinued the trial due to an episode of feeding line sepsis and malnutrition which resulted in her death 2 months after the last intake of drug.

No clinically relevant observations were made in clinical laboratory parameters, vital signs or electrocardiograms. Specifically there were no changes in QT duration with prucalopride treatment.

## Discussion

A large number of studies have demonstrated previously that prucalopride is effective in relieving symptoms in patients with chronic constipation.^11^–^15^ In particular, prucalopride increases bowel frequency and reduces abdominal bloating and pain.^11^ This study has shown that in some patients with chronic intestinal pseudo-obstruction prucalopride is effective in alleviating symptoms. To our knowledge this is the first long-term, double-blind, placebo-controlled, randomised study to show therapeutic benefit from medication in this disabling condition. Specifically, it was most effective in relieving the symptom of bloating. There are few, if any, options for managing this symptom.^16^ Prucalopride was also shown to be effective in reducing abdominal pain and nausea. Prucalopride reduced the frequency and severity of these symptoms.

Prucalopride is a potent, highly-selective 5HT_4_ agonist with no 5HT_3_ antagonism and no anticholinergic activity.^17^ It enhances peristalsis and accelerates colonic transit.^10^, ^18^–^20^ Prucalopride also accelerates small bowel transit in animals and humans.^10^, ^11^, ^18^ As such, prucalopride is a potentially beneficial agent in patients with CIP, where small and large bowel motility are perturbed. Doses of 1–4 mg of prucalopride have been shown to be effective in relieving the symptoms of constipation.^11^, ^12^ Given the severity of the condition, we chose to use a dose of 2–4 mg per day in this study.

An *n* = 1 cross-over study design potentially establishes the beneficial effects of a drug in an individual, in contrast to parallel-group studies which establish efficacy in a group of patients with a common condition.^21^ Given the fluctuating nature of symptoms in CIP it was important to have a long enough period of observation on both active drug and placebo. We reasoned that a 6-month exposure to each would permit this, spread over a year, with a randomised alternation between active drug and placebo.

An example of the impact of a single individual affecting the data set in a traditional group placebo-controlled study vs. their impact in an *n* = 1 study, such as this one, can be drawn from the symptoms experienced by patient 3006. This patient experienced major frequent symptoms in the year prior to entering this study, but few symptoms during the year of the study. This patient would have diluted the therapeutic assessment in a traditional study.

Prucalopride did not affect stool frequency and consistency. None of the four patients showed evidence of a major prokinetic effect on gastrointestinal transit. However, prucalopride did have an effect on gut sensory symptoms, improving nausea and pain. We have previously shown that prucalopride heightens visceral sensation to both distension and mucosal electric stimulation in patients with chronic constipation.^11^

Three subjects reported adverse events, but none were judged by the investigator, who was blind to the phase of treatment, to be related to study medication. Equally, no significant changes in haematology or biochemistry were identified following 6 months exposure to prucalopride. No electrocardiographical changes were seen, supporting previous studies showing that, unlike some other serotonergic compounds, prucalopride does not cause repolarisation changes.

In summary, prucalopride represents a potentially useful novel therapy for some patients with chronic intestinal pseudo-obstruction. The main benefit related to the relief of bloating and abdominal pain. The drug is well tolerated in this condition.
